# Transmission of Rift Valley fever virus from European-breed lambs to *Culex pipiens* mosquitoes

**DOI:** 10.1371/journal.pntd.0006145

**Published:** 2017-12-27

**Authors:** Rianka P. M. Vloet, Chantal B. F. Vogels, Constantianus J. M. Koenraadt, Gorben P. Pijlman, Martin Eiden, Jose L. Gonzales, Lucien J. M. van Keulen, Paul J. Wichgers Schreur, Jeroen Kortekaas

**Affiliations:** 1 Department of Virology, Wageningen Bioveterinary Research, Lelystad, the Netherlands; 2 Laboratory of Entomology, Wageningen University, Wageningen, the Netherlands; 3 Laboratory of Virology, Wageningen University, Wageningen, the Netherlands; 4 Institute of Novel and Emerging Infectious Diseases, Friedrich-Loeffler-Institut, Greifswald—Insel Riems, Germany; 5 Department of Bacteriology and Epidemiology, Wageningen Bioveterinary Research, Lelystad, the Netherlands; INDEPENDENT RESEARCHER, UNITED STATES

## Abstract

**Background:**

Rift Valley fever virus (RVFV) is a mosquito-borne bunyavirus of the genus *Phlebovirus* that is highly pathogenic to ruminants and humans. The disease is currently confined to Africa and the Arabian Peninsula, but globalization and climate change may facilitate introductions of the virus into currently unaffected areas via infected animals or mosquitoes. The consequences of such an introduction will depend on environmental factors, the availability of susceptible ruminants and the capacity of local mosquitoes to transmit the virus. We have previously demonstrated that lambs native to the Netherlands are highly susceptible to RVFV and we here report the vector competence of *Culex (Cx*.*) pipiens*, the most abundant and widespread mosquito species in the country. Vector competence was first determined after artificial blood feeding of laboratory-reared mosquitoes using the attenuated Clone 13 strain. Subsequently, experiments with wild-type RVFV and mosquitoes hatched from field-collected eggs were performed. Finally, the transmission of RVFV from viremic lambs to mosquitoes was studied.

**Principal findings:**

Artificial feeding experiments using Clone 13 demonstrated that indigenous, laboratory-reared *Cx*. *pipiens* mosquitoes are susceptible to RVFV and that the virus can be transmitted via their saliva. Experiments with wild-type RVFV and mosquitoes hatched from field-collected eggs confirmed the vector competence of *Cx*. *pipiens* mosquitoes from the Netherlands. To subsequently investigate transmission of the virus under more natural conditions, mosquitoes were allowed to feed on RVFV-infected lambs during the viremic period. We found that RVFV is efficiently transmitted from lambs to mosquitoes, although transmission was restricted to peak viremia. Interestingly, in the mosquito-exposed skin samples, replication of RVFV was detected in previously unrecognized target cells.

**Significance:**

We here report the vector competence of *Cx*. *pipiens* mosquitoes from the Netherlands for RVFV. Both laboratory-reared mosquitoes and well as those hatched from field-collected eggs were found to be competent vectors. Moreover, RVFV was transmitted efficiently from indigenous lambs to mosquitoes, although the duration of host infectivity was found to be shorter than previously assumed. Interestingly, analysis of mosquito-exposed skin samples revealed previously unidentified target cells of the virus. Our findings underscore the value of including natural target species in vector competence experiments.

## Author summary

The consequences of first introductions of mosquito-borne viruses into previously unaffected areas depend on environmental factors, the availability of susceptible hosts and local vector populations. We have previously demonstrated that sheep breeds native to the Netherlands are highly susceptible to Rift Valley fever virus (RVFV), a mosquito-borne virus that causes severe outbreaks among domesticated ruminants and humans in Africa and the Arabian Peninsula. To gain further insight into the risk of a future RVFV introduction into the Netherlands, we have now investigated the vector competence of *Cx*. *pipiens*, the most abundant mosquito species in the country. Vector competence was first determined after artificial blood feeding and subsequently after feeding on viremic lambs. The results from artificial feeding experiments suggested that indigenous *Cx*. *pipiens* mosquitoes are competent vectors. The vector competence of *Cx*. *pipiens* was confirmed after feeding on viremic lambs. Transmission from lambs to mosquitoes was found to be very efficient, although largely confined to peak viremia. The localized inflammatory response resulting from mosquito bites was associated with enhanced virus replication in the skin.

## Introduction

RVFV is a mosquito-borne zoonotic bunyavirus that predominantly affects domesticated and wild ruminants. Near simultaneous abortions of gestating sheep and high numbers of newborn lamb fatalities are characteristic features of RVF outbreaks. Human infections generally result in a self-resolving, acute and febrile illness, although a small percentage of infected individuals develop more severe complications including retinopathy, encephalitis or hemorrhagic fever, the latter with often fatal outcome. Since its discovery in the 1930s, the virus has spread across the African continent and invaded the Arabian Peninsula and several islands off the coast of Southeast Africa [1,2]. The worldwide distribution of mosquito species that are associated with transmission in endemic areas raises concerns that RVFV may follow in the footsteps of West Nile virus, chikungunya virus and Zika virus.

RVFV has been isolated from over 30 species of mosquitoes (Diptera: *Culicidae*) belonging to 10 different genera [3]. Many of these mosquito species were found capable of transmitting the virus, at least under experimental conditions [2]. A landmark study by Linthicum and co-workers demonstrated that RVFV can be transmitted vertically to the eggs of the mosquito species *Aedes* (*Neomelaniconion*) *mcintoshi* Huang, a species that was misidentified and cited before 1985 as *Aedes lineatopennis* [4,5]. This mosquito is known as a “floodwater” mosquito, as females of this species deposit eggs near depressions that seasonally flood, known as “pans” or “dambos” in endemic areas. The eggs need to dehydrate before they can hatch upon rehydration. Eggs of such floodwater mosquitoes can survive long periods of drought, possibly contributing to the persistence of RVFV during long interepidemic periods.

Upon hatching of infected mosquito eggs, the infected females may transmit the virus to susceptible animals during blood feeding. The virus may circulate between floodwater mosquitoes and ruminants at low level in sylvatic cycles for many years without causing epizootics. In those periods, human cases occur sporadically, as floodwater mosquitoes are generally zoophilic. After periods of above-average rainfall, mosquito populations can increase dramatically. Various alternative species of mosquitoes may then contribute to transmission of the virus, including anthropophilic mosquito species that may introduce the virus into the human population. When such mosquito species become abundant, large outbreaks among humans may follow [1,2].

One of the largest “virgin soil” epidemics of RVF occurred in Egypt in 1977–78 [6]. This outbreak followed the completion of the Aswan High Dam across the Nile river, which resulted in new permanent fresh water breeding sites for mosquitoes. During this outbreak, millions of animals and an estimated 200,000 humans became infected with the virus. Soon after the Egyptian outbreak, two studies reported that almost all mosquitoes collected from the affected areas of the Nile delta belonged to the *Cx*. *pipiens* complex [7,8]. These mosquitoes were subsequently shown to transmit the virus to susceptible hamsters, thereby confirming that *Cx*. *pipiens* is a competent vector of RVFV, at least in these regions [7,8]. Further studies have demonstrated that *Cx*. *pipiens* mosquitoes from other areas, including the US and Europe, are capable of transmitting RVFV, although significant differences in competence may exist between vectors collected from different areas due to (epi)genetic and environmental factors [9–16]. Consequently, to assess the risk of RVFV outbreaks in currently unaffected areas, insight into the vector competence of local vector populations is crucial.

*Cx*. *pipiens* is the most abundant and widespread mosquito species in several European countries, including the Netherlands [17,18]. To assess the risk of a future RVFV outbreak in the Netherlands, our laboratory has previously evaluated the susceptibility of indigenous sheep breeds [19–21]. We have now continued our risk-assessment with studies on the vector competence of local *Cx*. *pipiens* mosquitoes. To facilitate our initial experiments, we made use of the attenuated Clone 13 strain, which can be handled safely in biosafety level-2 (BSL-2) laboratories and was previously used successfully for RVFV vector competence studies [10,12]. After initial experiments with Clone 13 and laboratory-reared mosquitoes, the vector competence of indigenous *Cx*. *pipiens* mosquitoes was confirmed by experiments with wild-type RVFV and mosquitoes hatched from field-collected eggs. Finally, the efficiency of transmission from viremic lambs to mosquitoes was investigated. Our results show that *Cx*. *pipiens* mosquitoes of the Netherlands are competent vectors of RVFV and that the virus is efficiently transmitted from indigenous lambs to mosquitoes.

## Materials and methods

### Virus and cells

Culture media and supplements were obtained from Gibco unless indicated otherwise. C6/36 (*Aedes albopictus*) cells (ATCC CRL-1660) were cultured at 28°C in absence of CO_2_ in L15 medium (Sigma) supplemented with 10% fetal bovine serum (FBS, Bodinco), 2% Tryptose Phosphate Broth (TPB), and 1% MEM nonessential amino acids solution (MEMneaa). Vero cells (ATCC CRL-1586) were cultured in DMEM GlutaMAX supplemented with 3% FBS, 1% Pen/Strep, and 1% Fungizone (DMEM^+^) at 37°C and 5% CO_2_.

The Clone 13 virus was generously provided by Institut Pasteur. Clone 13 is a plaque-purified clone, derived from strain 74HB59, which was isolated from a human case in the Central African Republic. Clone 13 contains a 69% deletion in the gene encoding the non-structural NSs protein, which was shown to counteract host innate immune responses via several mechanisms and is thereby considered the major virulence determinant [22–27]. Indeed, viruses that lack NSs expression, such as Clone 13, are highly attenuated in mice [27], sheep [28], and cattle [29].

Wild-type recombinant strain 35/74 is derived from strain 35/74 that was isolated from the liver of a sheep that died during an RVFV outbreak in the Free State province of South Africa in 1974. The original virus was passaged in suckling mouse brain four times and subsequently four times in BHK cells. The full genome sequence of this virus was used to synthesize cDNA. Full genome sequences of this virus were deposited in GenBank under the accession numbers JF784386, JF784387, and JF784388. The virus was rescued using BSR-T7 cells and passaged once in BHK cells [30]. Recombinant 35/74 virus is highly virulent for sheep, as demonstrated in our previous studies [19–21].

Clone 13 and strain 35/74 were amplified in C6/36 cells, starting with a multiplicity of infection of 0.005. Culture medium was harvested after 4 days and cleared by slow-speed centrifugation. Virus titers were determined by end point dilution assay as 50% tissue culture infective dose per ml (TCID_50_/ml). Briefly, Vero cells were seeded in 96 well plates at an approximate density of 20,000 cells/well in 100 μl DMEM^+^. The next day, 10-fold dilutions of culture media containing virus were added to each well (50μl/well). After 4–5 days incubation, the wells were scored for cytopathic effect. Titers were determined as TCID_50_/ml using the Spearman-Kärber algorithm [31,32].

For blood feeding experiments, blood was collected from cattle of Wageningen Bioveterinary Research (WBVR, Lelystad, the Netherlands). Erythrocytes were collected from freshly collected EDTA blood by slow-speed centrifugation (650 *xg*) and washed three times with PBS. Washed erythrocytes were subsequently resuspended in L15 complete medium (L15 + 10% FBS, 2% TPB, 1% MEMneaa) to a concentration that is four times higher than found in blood. To prepare a blood meal, one part of the erythrocyte suspension was mixed with two parts of culture medium containing virus.

### General procedures of artificial mosquito feeding, forced salivation and virus isolation

Laboratory-reared *Cx*. *pipiens* mosquitoes were provided by the Laboratory of Entomology of Wageningen University. These mosquitoes were previously shown to be competent vectors of West Nile virus and Usutu virus [33,34]. The mosquitoes were reared from an above-ground collected pool of egg rafts originating from Brummen, the Netherlands. The colony was established in 2010 and maintained at 23°C in BugDorm cages with a 16:8 h light:dark cycle and 60% humidity. The mosquitoes were provided with 6% glucose for general maintenance and with bovine (Carus, Wageningen, the Netherlands) or chicken (Kemperkip, Uden, the Netherlands) blood for egg production using a Hemotek PS5 feeder (Discovery Workshops). Egg rafts were allowed to hatch in tap water supplemented with Liquifry No. 1 (Interpet Ltd.). Larvae were fed with a mixture (1:1:1) of bovine liver powder, ground koi carp food, and ground rabbit food. In addition to this mosquito colony, we made use of *Cx*. *pipiens* mosquitoes hatched from eggs collected in Best, the Netherlands, and subsequently treated similarly as the colonized mosquitoes.

Prior to artificial feeding experiments, mosquitoes were transported to Wageningen Bioveterinary Research (WBVR) and allowed to acclimatize for 3 days in an insect incubator (KBWF 240, Binder) at 23°C at a humidity of 70% and a 16:8 h light:dark cycle. Feeding was performed in white buckets (1L) covered with mosquito netting, using a Hemotek PS5 feeder [33]. Of note, feeding with Clone 13 was performed inside the insect incubator, whereas feeding with virulent wild-type strain 35/74 was performed in a class-III biosafety cabinet (glovebox). After feeding on blood meals containing different amounts of virus as described for individual experiments in more detail below, fully engorged mosquitoes were collected with an automatic insect aspirator and maintained with sugar water (6% sucrose solution), provided in flasks with filter paper. After the required incubation periods, mosquitoes were sedated on a semi-permeable CO_2_-pad connected to 100% CO_2_. Prior to the salivation assay, wings and legs were removed. Saliva was collected by forced salivation using 20 μl filter tips containing 7 μl of a 1:1 mixture of FBS and 50% sucrose (capillary tube method). After 1–1.5h, saliva samples were collected and incubated with Vero cell monolayers. After 3 h incubation at 37°C, the inocula were replaced by fresh medium. Cytopathic effect (CPE) was scored 3–5 days later.

Mosquito bodies were initially stored at -80°C. For analysis, bodies were thawed and homogenized in 500 μl DMEM+ with a pellet pestle (Sigma) and the homogenate was subsequently cleared by slow speed centrifugation. Part of the material (70μl) was used for virus isolation using the virus titration protocol described above. Another proportion of the material (200μl) was used for RVFV specific quantitative reverse-transcriptase PCR (qRT-PCR) as described previously [35].

The percentage of blood fed mosquitoes that contained virus after the incubation period as demonstrated by virus isolation from the mosquito body was defined as the infection rate. The transmission rate was defined as the percentage of blood fed mosquitoes that contained virus in their saliva after the incubation period.

### Influence of virus dose on infection and transmission rates

In Experiment 1, laboratory-reared mosquitoes were fed with bovine erythrocyte suspensions, prepared as described above, spiked with 10^5.3^ (low dose), 10^7.3^ (medium dose) or 10^9.3^ (high dose) TCID_50_/ml of freshly prepared Clone 13 virus. Virus titers were confirmed retrospectively. Mosquitoes were subsequently maintained for 21 days at 28°C after which bodies and saliva samples were collected and used for virus isolation.

### Vector competence of *Cx*. *pipiens* hatched from field-collected eggs

To investigate the vector competence of mosquitoes hatched from field-collected eggs, mosquitoes were fed with bovine erythrocyte suspensions containing a dose of 10^9.3^ TCID_50_/ml of Clone 13 (Experiment 2) or with blood meals containing 10^8.0^ TCID_50_/ml of Clone 13 or virulent strain 35/74 (Experiment 3). To enable the use of similar titers for the comparison of Clone 13 and strain 35/74, virus batches were prepared, titrated, set to equal titers and stored at -80°C until use. Mosquitoes were maintained for 14 days at 28°C, after which the infection and transmission rates were determined.

### Feeding of mosquitoes on viremic lambs

To determine the infectious period of lambs for *Cx*. *pipiens* mosquitoes (Experiment 4), two 12-week-old Dutch lambs (Texel/Swifter) were inoculated, under BSL-3 conditions, with 10^5.0^ TCID_50_/ml of the highly virulent RVFV strain 35/74 via intravenous route. EDTA blood samples, to be used for RVFV qRT-PCR and virus isolation, were collected every day after challenge. On days, 1, 2, 3, and 4 post inoculation, cardboard cups containing 50 female, laboratory-reared *Cx*. *pipiens* mosquitoes were placed on the inner thigh of a hind leg of the sheep. A single feeding site was used on each animal. The wool was removed from this part of the body using Veet hair removal cream, and the cup was fixed with elastic bandage. After 45 min the cups with mosquitoes were removed and brought to the BSL-3 laboratory. The mosquitoes were maintained for 7 days at 20°C and subsequently transferred to 28°C. Bodies and saliva samples were collected after 5–7 days at 28°C and analysed for the presence of virus by qRT-PCR and virus isolation as described above.

### Histopathology and immunohistochemistry

After death or euthanasia of the two RVFV infected lambs, tissue samples obtained from the liver, spleen, adrenal gland and hepatic lymph node were collected for histopathological examination. In addition, the skin was sampled both from the site affected by the mosquito bites and from the unbitten skin of the opposite leg. Tissue samples were fixed for 48 h in 10% phosphate buffered formalin and processed routinely into paraffin blocks. Sections were cut on silane-coated glass slides and dried for at least 48 h in a 37°C incubator. Sections were stained routinely with haematoxylin and eosin (H&E) or immunostained for RVFV antigen. Briefly, endogenous peroxidase was blocked for 30 min in methanol/H_2_O_2_ followed by enzymatic digestion in 0.1% trypsin (Difco Laboratories) to retrieve relevant epitopes. As primary antibody, monoclonal antibody (mAb) 9 was used, which recognizes the nucleocapsid protein [36]. Mouse Envision peroxidase and DAB+ chromogen (Dakopatts, Denmark) were used as substrate, according to the manufacturer’s instructions.

### Ethics statement

All animal experiments were conducted in accordance with the Dutch Law on Animal Experiments (Wet op de Dierproeven, ID number BWBR0003081) and were approved by the Animal Ethics Committee of Wageningen Bioveterinary Research (WBVR), in accordance with the regulations of EU directive 2010/63/EU and the Experiments on Animals Act, 1997. To minimize suffering of the animals from the RVFV infection, lambs were euthanized when they reached a predefined humane endpoint.

### Statistical analyses

Statistical significance of the effect of the dose of ingested virus on virus titers in mosquito bodies after the incubation period was calculated using the Mann-Whitney test and differences between infection and transmission rates were calculated using Fisher’s exact test. The differences in virus titers in the bodies of mosquitoes after feeding on viremic lambs and the corresponding transmission rates were also calculated with the Mann-Whitney test and Fisher’s exact test, respectively. The threshold for significance was adjusted by applying the Bonferroni correction p < 0.05/n, where n was the number of between group comparisons. These analyses were performed using GraphPad 6.

## Results

### Influence of virus dose on infection and transmission rates

**Fig 1:**
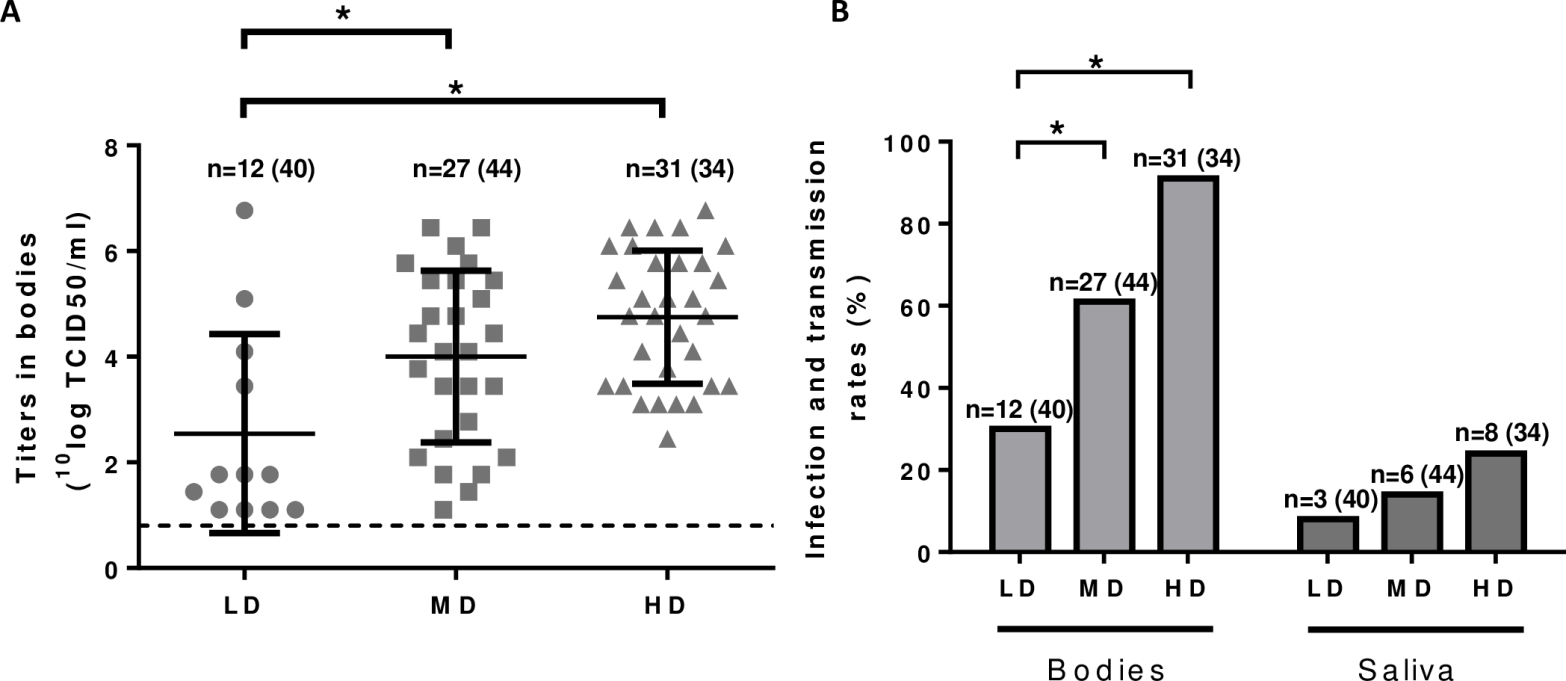
Influence of virus dose on infection and transmission rates. Mosquitoes were allowed to feed on a suspension of bovine erythrocytes containing a low dose (LD, 10^5.3^ TCID_50_/ml), medium dose (MD, 10^7.3^ TCID_50_/ml) or high dose (HD, 10^9.3^ TCID_50_/ml) of RVFV Clone 13 and were maintained for 21 days at 28°C. (A) Symbols represent virus titers in the bodies of the mosquitoes that were found virus positive after the incubation period. Means with SDs (error bars) and the detection limit of the virus isolation assay (dashed line) are indicated. (B) Infection and transmission rates. Asterisks indicate statistically significant differences (P<0.017) as determined using the Mann-Whitney test (panel A) or Fisher’s exact test (panel B).

To determine if *Cx*. *pipiens* mosquitoes native to the Netherlands are susceptible to RVFV infection and to study the effect of virus dose (Table 1, Experiment 1), mosquitoes were fed with bovine erythrocyte suspensions spiked with different doses of Clone 13. Virus titers in the bodies that were found positive after the incubation period are depicted in Fig 1A. The infection and transmission rates are depicted in Fig 1B. Mosquitoes from the low-dose group revealed an infection rate of 30% and a transmission rate of 8%, whereas those from the medium-dose group revealed an infection rate of 64% and a transmission rate of 14%. The infection and transmission rates in the high dose group were 74% and 24%, respectively (Fig 1B and Table 1). These results indicate that *Cx*. *pipiens* mosquitoes from the Netherlands are competent to transmit RVFV and that infection rates increase with the dose of ingested virus. The effects of virus dose on transmission rates were not statistically significant, probably due to the low number of mosquitoes with infectious virus in their saliva.

**Table 1:** Infection and dissemination rates in *Cx*. *pipiens* mosquitoes after oral exposure to Clone 13 or wild-type RVFV strain 35/74^a^.

Exp. no:	Mosquito origin:	Feeding method:	Day of feeding on lambs:	Virus:	Titer^a^:	Incubation temp (°C)^b^:	Time (DPF)^c^:	N:	IR (%):	TR (%):
1	Laboratory reared	Hemotek	*N*.*A*.	Clone 13	5.3	28	14	40	30	8
					7.3		14	44	64	14
					9.3		14	34	74	24
2	Field collected	Hemotek	*N*.*A*.	Clone 13	9.3	28	14	40	60	18
3	Field collected	Hemotek	*N*.*A*.	Clone 13	8.0	28	14	45	24	2
				35/74			14	20	60	25
4	Laboratory reared	Lamb 1	1	35/74	3.0	20°C+28°C	7+7	14	0	0
			2		6.4		7+6	23	91	30
			3		5.7		7+6	11	18	0
			4		4.5		7+5	11	18	0
		Lamb 2	1		2.5		7+7	4	0	0
			2		5.2		7+6	7	86	29
			3		4.2		7+6	9	11	11

The most important variables in each experiment are shaded.

^a^Titers are depicted as ^10^log TCID_50_/ml.

^b^Mosquitoes were either maintained at one temperature or first at 20°C and subsequently at 28°C.

^c^When mosquitoes were incubated at two temperatures, the first number represents days incubated at 20°C and the second number the days incubated at 28°C.

DPF, days post feeding; N, Number of assayed mosquitoes; IR, infection rate; TR, transmission rate; N.A., Not Applicable.

### Vector competence of *Cx*. *pipiens* hatched from field-collected eggs

**Fig 2:**
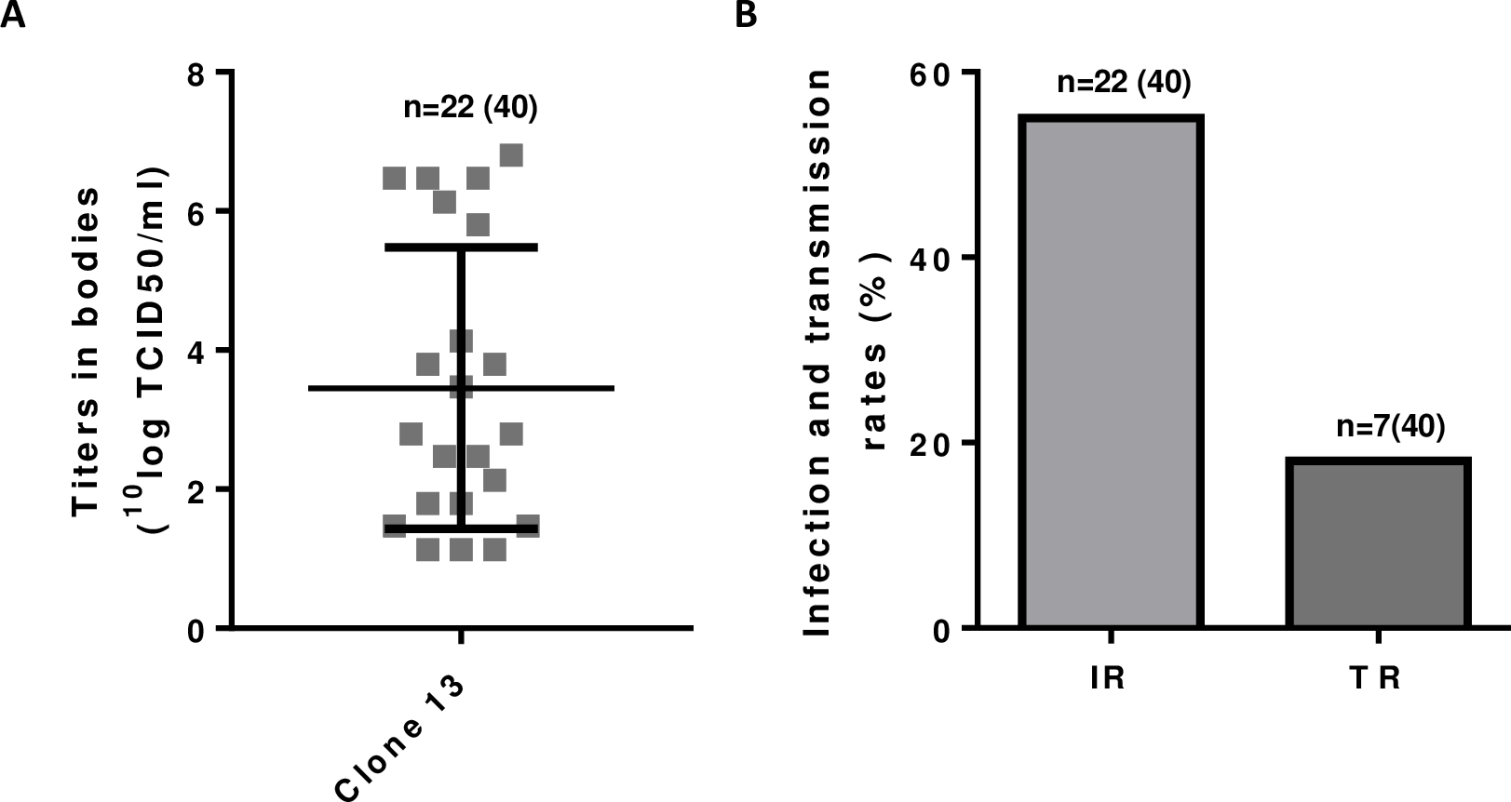
Vector competence of *Cx*. *pipiens* hatched from field-collected mosquito eggs. Mosquitoes hatched from field-collected eggs were allowed to feed on a blood meal containing 10^9.3^ TCID_50_/ml of Clone 13. After incubation for 14 days at 28°C, the titers in mosquito bodies (A) and infection and transmission rates (B) were determined.

To confirm the vector competence of Dutch *Cx*. *pipiens* mosquitoes for RVFV, mosquitoes hatched from field-collected eggs were fed with a blood meal containing 10^9.3^ TCID_50_/ml of Clone 13. The virus titers in bodies and infection and transmission rates are depicted in Fig 2. Although the experiments were not performed at the same time, feeding with a dose of 10^9.3^ TCID_50_/ml of Clone 13 in Experiment 1 resulted in an infection rate of 74% after incubation at 28°C for 14 days, whereas feeding the same dose to mosquitoes hatched from field-collected eggs in the present experiment resulted in an infection rate of 60%. Transmission rates were also somewhat lower in the latter: 18% versus 24% (Table 1).

**Fig 3:**
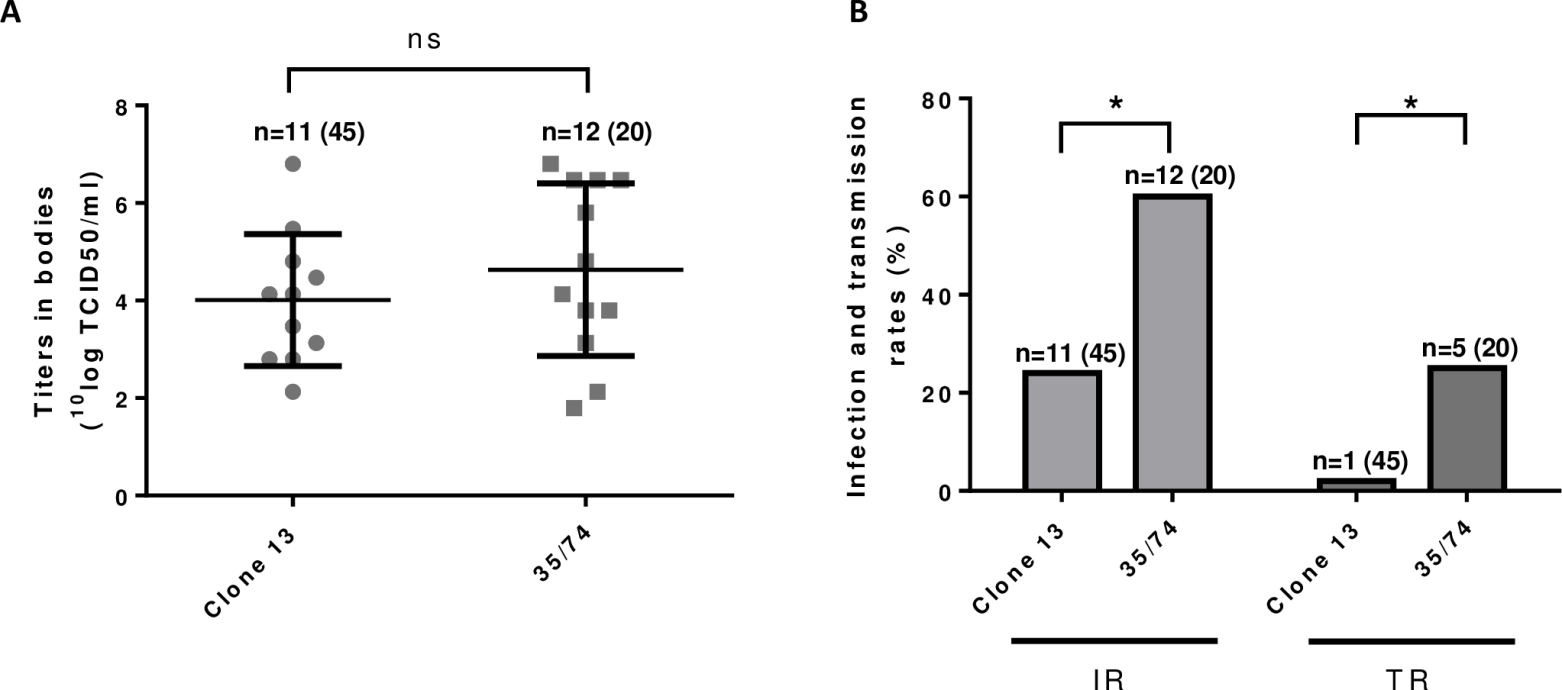
Comparing the vector competence of *Cx*. *pipiens* hatched from field-collected mosquito eggs for Clone 13 and wild-type strain 35/74. Mosquitoes hatched from field-collected eggs were allowed to feed on a blood meal containing 10^8.0^ TCID_50_/ml of Clone 13 or virulent strain 35/74. After incubation for 14 days at 28°C, the titers in mosquito bodies (A) and infection and transmission rates (B) were determined. Asterisks indicate statistically significant differences (P<0.05).

In Experiment 3, *Cx*. *pipiens* mosquitoes hatched from field-collected eggs were fed with a blood meal containing 10^8.0^ TCID_50_/ml of Clone 13 or virulent strain 35/74. After incubation for 14 days at 28°C, the titers in virus-positive bodies were found to be comparable between mosquitoes fed with Clone 13 and strain 35/74 (Fig 3A), whereas both infection and transmission rates were significantly higher in mosquitoes fed with strain 35/74 (Table 1 and Fig 3B). These results confirm that *Cx*. *pipiens* mosquitoes hatched from eggs collected in the Netherlands are competent vectors of wild-type RVFV.

### Transmission of RVFV from viremic lambs to *Cx*. *pipiens* mosquitoes

**Fig 4:**
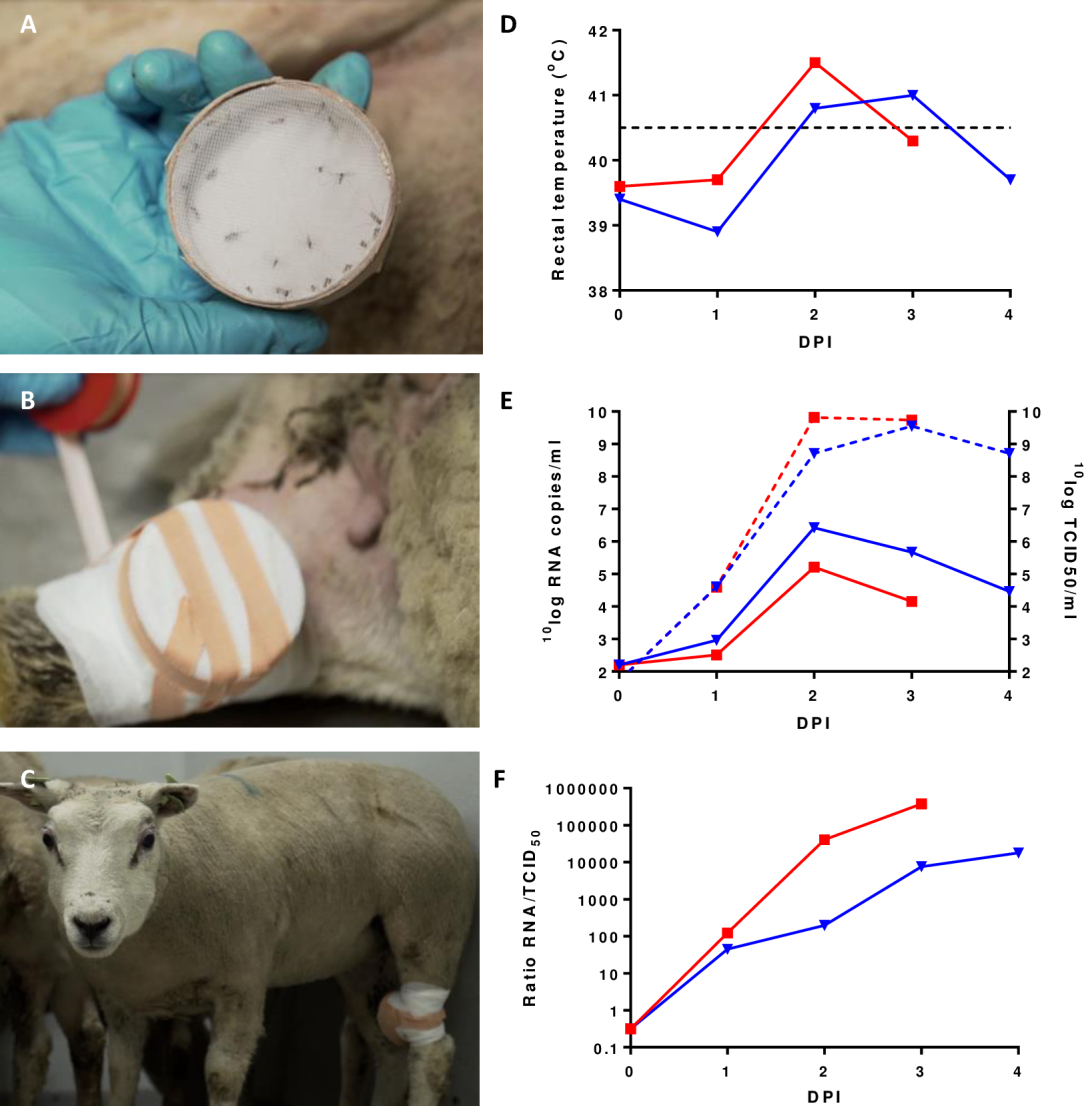
Feeding of *Cx*. *pipiens* mosquitoes on lambs during RVFV viremia. Two lambs were inoculated via intravenous route with the highly virulent 35/74 strain. Groups of 50 mosquitoes were allowed to feed on the lambs every following day. The method used to place containers on the lambs is depicted in panels A-C. Both lambs developed fever on DPI 2 (D). Lamb 1 (▼) was euthanized on DPI 4, whereas lamb 2 (■) succumbed to the infection on DPI 3. E. Levels of viral RNA (dashed lines) and infectious virus (solid lines) in plasma samples of the lambs. F. Ratios of viral RNA and infectious virus during viremia.

To determine if Dutch *Cx*. *pipiens* mosquitoes become infected after feeding on viremic lambs, two lambs were inoculated intravenously with 10^5^ TCID_50_/ml RVFV strain 35/74. Every following day, groups of 50 female mosquitoes were allowed to obtain a blood meal. The procedure is visualized in Fig 4A–4C.

Both lambs developed fever (Fig 4D) and high viremia on the second day post infection (DPI 2) as determined by qRT-PCR and virus isolation (Fig 4E). One lamb succumbed to the infection on DPI 3, whereas the other was euthanized on DPI 4 after reaching a humane end-point. This lamb was euthanized by exsanguination, after being anesthetized with 50 mg/kg sodium pentobarbital (EuthasolH, ASTfarma BV, The Netherlands) applied via the intravenous route. Post-mortem analysis revealed massive hepatic necrosis in both lambs, which is characteristic of a fatal outcome of RVFV infection.

Some engorged mosquitoes were found dead upon arrival in the BSL-3 laboratory. To maintain adequate group sizes, we decided to maintain the mosquitoes at 20°C instead of 28°C during the first 7 days. To stimulate virus dissemination, the mosquitoes were subsequently placed at 28°C. Although it was our intention to maintain the mosquitoes for 7 days at 28°C, we decided to sample some of the groups earlier to maintain adequate sample sizes (Table 1).

**Fig 5:**
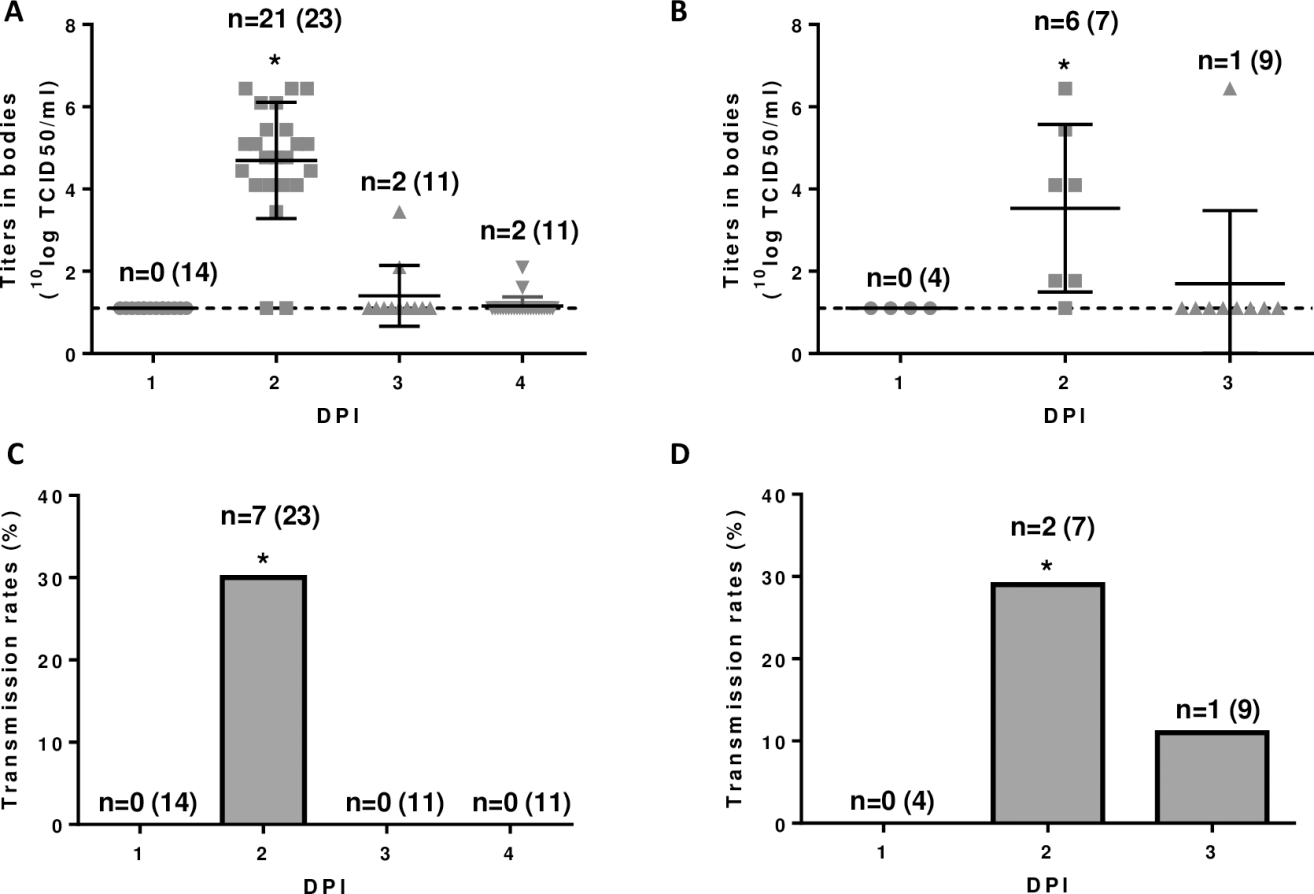
Transmission of RVFV from viremic lambs to *Cx*. *pipiens* mosquitoes. Mosquitoes were allowed to feed on lambs at days 1–4 post infection (DPI). Virus titers in the bodies of the mosquitoes after the incubation period are shown in panels A (mosquitoes that fed on lamb 1) and B (mosquitoes that fed on lamb 2). Means with SDs and the detection limit of the virus isolation assay are indicated. The transmission rates are shown in panels C (mosquitoes that fed on lamb 1) and D (mosquitoes that fed on lamb 2). Asterisks indicate statistically significant differences (P<0.01) between the indicated group and the other groups as determined using the Mann-Whitney test (A and B) or Fisher’s exact test (C and D).

Surprisingly, the infection rates were very high (86–91%) in the groups of mosquitoes fed on DPI 2, whereas relatively low infection rates (11–18%) were detected in the groups of mosquitoes fed on DPI 3 (Fig 5A and 5B). This was particularly surprising as viral RNA levels in the blood were equally high, or even higher on DPI 3 (Fig 4E). However, virus isolation demonstrated that infectious virus titers in the blood were higher on DPI 2 than on DPI 3 (Fig 4E). Apparently, non-infectious virus or viral RNA rapidly accumulated in the blood of the lambs between DPI 2 and 3, which can be visualized by the ratios of viral RNA levels and infectious virus titers (Fig 4F). Importantly, the lower infection rates (Fig 5A and 5B) correlated well with transmission rates, being 29–30% in mosquitoes that fed on DPI 2 and 0–11% in mosquitoes fed on DPI 3 (Fig 5C and 5D). These results suggest that efficient transmission from viremic lambs to mosquitoes is largely limited to peak viremia.

### Increased virus replication at mosquito feeding sites

**Fig 6:**
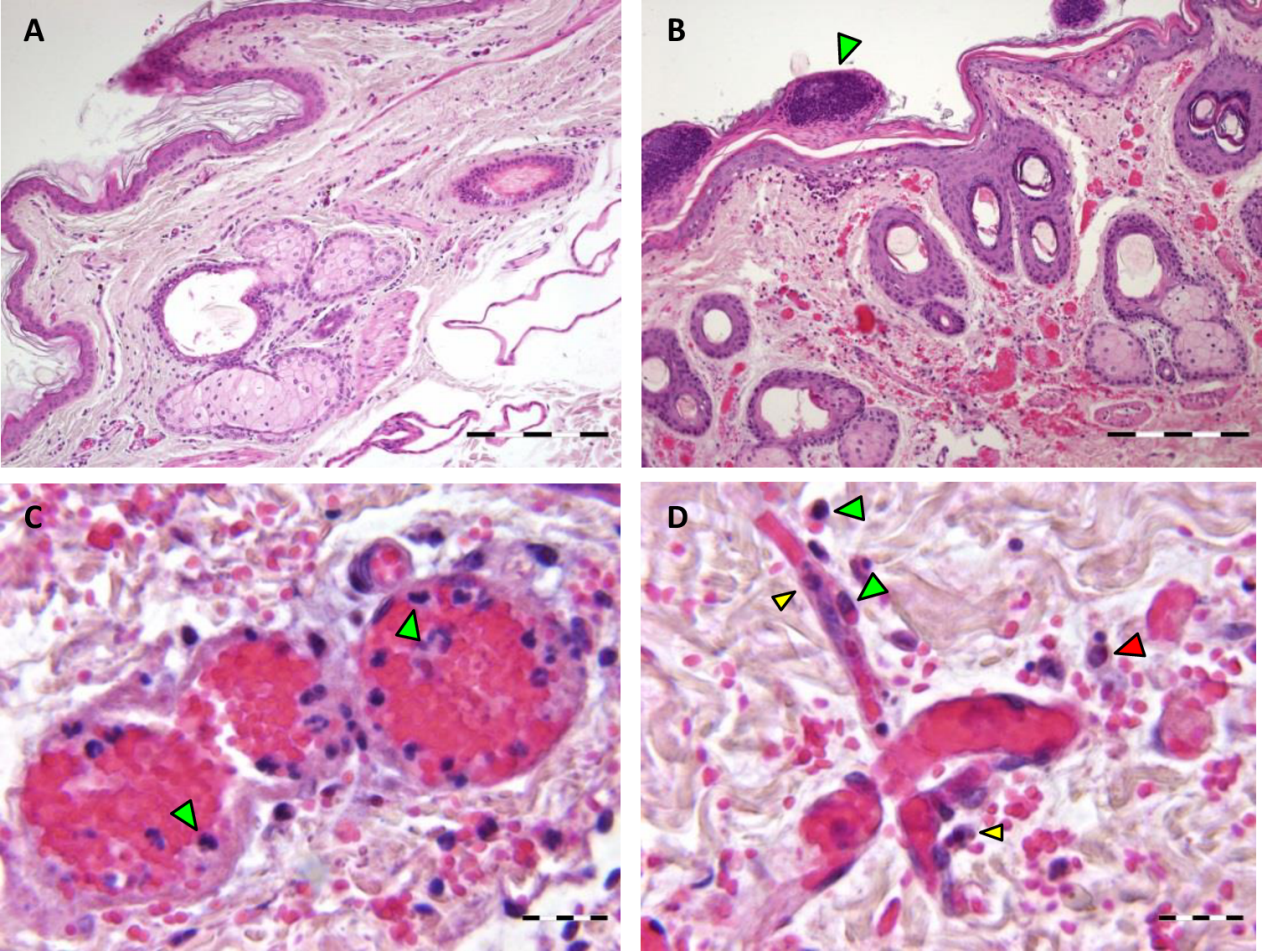
Hematoxylin and eosin staining of unexposed (A) and mosquito-exposed (B) skin. Staining of mosquito-exposed skin reveals dilatation of the blood vessels with extensive haemorrhages in the dermis. Hydropic degeneration of keratinocytes in the epidermis, exocytosis of neutrophils and crust formation (green arrowhead). (C) Margination of neutrophils and thrombocytes in capillaries and venules (green arrowheads). (D) Influx of neutrophils (yellow arrowheads) and macrophages (green arrowheads) into the dermis. Macrophages show phagocytosis of erythrocytes (green arrowheads) and an apoptotic neutrophil (red arrowhead). Bar = 200 μm (A, B), 20 μm (C, D).

Samples from unexposed skin (Fig 6A) and mosquito-exposed skin (Fig 6B) were examined by H&E staining and immunohistochemistry (IHC). H&E staining revealed extensive haemorrhages in the superficial and deep dermis at the site of mosquito bites (Fig 6B). Blood vessels were severely dilated centrally filled with erythrocytes while neutrophils and thrombocytes showed margination at the periphery of the blood vessels (Fig 6C). In the dermis, an increased influx of both neutrophils and macrophages was noticed compared to the unbitten skin of the opposite leg (Fig 6D). The epidermis showed hydropic degeneration of keratinocytes, acantholysis and cleft formation. Exocytosis of neutrophils was observed with crust formation on the epidermis (Fig 6B).

**Fig 7:**
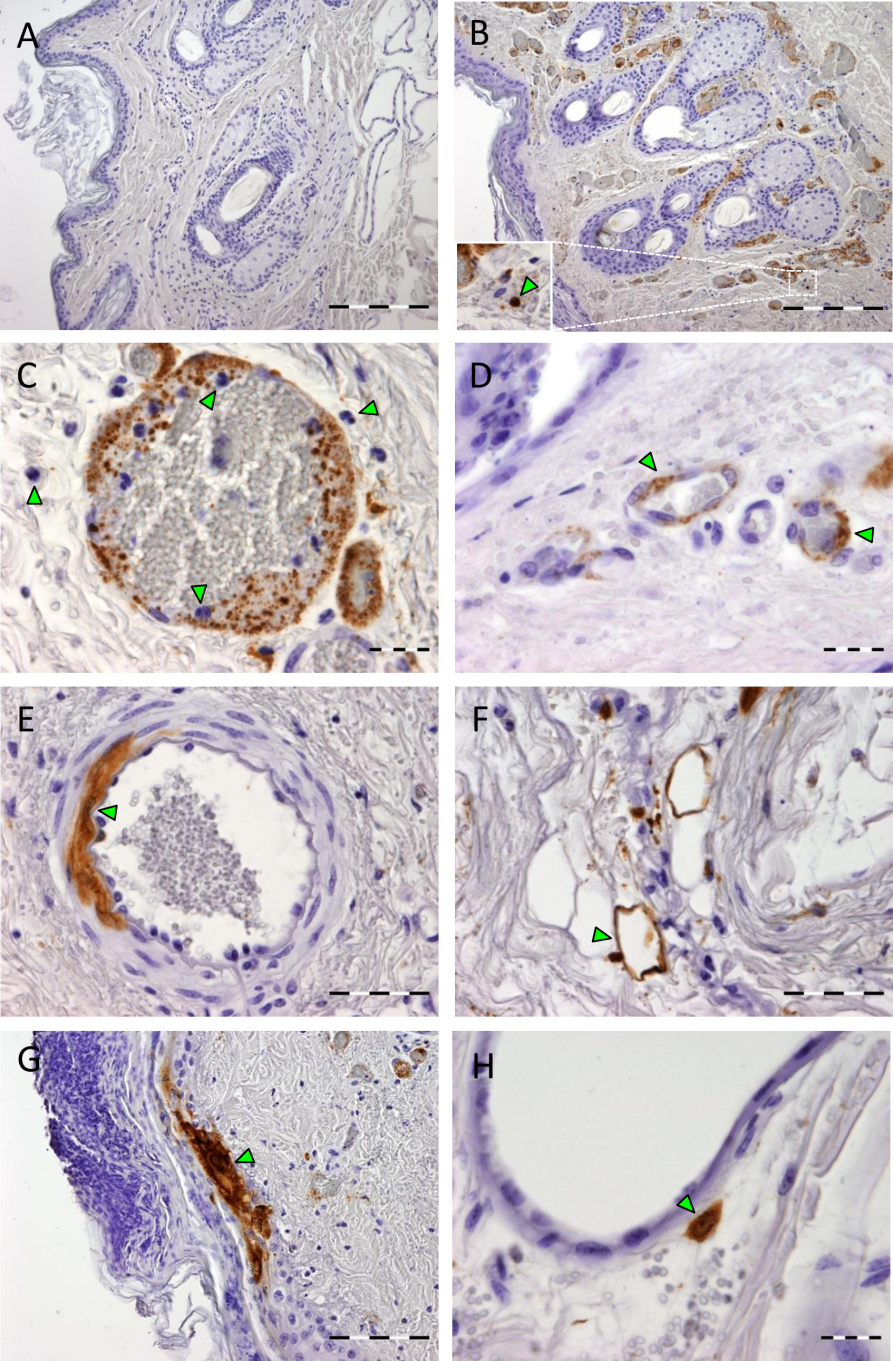
Increased virus replication in the skin of lambs after mosquito feeding. Immunohistochemical staining of RVFV nucleocapsid protein using mAb 9 as the primary antibody. Detection of viral antigen (brown) in mosquito unexposed- (A) or exposed (B) skin of the same animal. The inset in B shows staining of RVFV antigen in a macrophage. (C) Higher magnification of a blood vessel, showing margination of RVFV antigen together with neutrophils and thrombocytes with emigration of neutrophils). Notice the absence of viral staining in the cytoplasm of neutrophils (arrowheads). (D-H). positive staining (arrowheads) of endothelial cells (D), smooth muscle cells of the tunica media (E), lipocytes (F), keratinocytes (G) and fibroblasts (H). Bar = 200 μm (A, B), 100 μm (G), 50 μm (E, F) and 20 μm (C, D, H).

Immunostaining was performed with mAb 9 specific for the N protein (Fig 7). Analysis of mosquito-exposed skin revealed heavy staining of RVFV antigen in endothelial cells of dermal blood vessels, smooth muscle cells, lipocytes, keratinocytes and fibroblasts (Fig 7). Strikingly, RVFV antigen was associated with the margination of thrombocytes observed in the blood vessels (Fig 7C). RVFV antigen was detected in the cytoplasm of infiltrating macrophages but not in neutrophils (Fig 7A and 7C). In the epidermis, localized areas of positively stained keratinocytes were observed located in the stratum basale, stratum spinosum and stratum granulosum (Fig 7G). Importantly, no RVFV antigen was observed in skin samples obtained from the other leg of the same animal or in control sections of the skin of an uninfected sheep (Fig 7A).

## Discussion

To gain insight into the consequences of a potential future introduction of RVFV into the Netherlands, our laboratory previously investigated the susceptibility of indigenous sheep breeds. These studies demonstrated that local sheep breeds are highly susceptible to RVFV, resulting in mortality rates varying from 20% to 70% [19–21]. Here, we show that *Cx*. *pipiens* mosquitoes, the most abundant and widespread mosquito species in the Netherlands and elsewhere in Europe, are competent vectors of RVFV, as was previously demonstrated for *Cx*. *pipiens* mosquitoes from other areas [8–10,13,37–39]. Interestingly, results obtained from a direct comparison between Clone 13 and wild-type RVFV are in line with earlier indications that NSs contributes to replication in mosquitoes [13,14,40].

To evaluate whether the mosquitoes would be competent vectors under more natural conditions, *Cx*. *pipiens* mosquitoes were allowed to feed on viremic lambs during different stages of viremia. This resulted in high infection rates of 86–91% and transmission rates of 29–30%. As expected, most mosquitoes were infected after feeding during peak viremia, occurring on day 2 after infection of the lambs. It was, however, surprising to find that almost no transmission took place from viremic lambs to mosquitoes during the following days. Although viral RNA levels were comparable the day after peak viremia, virus isolation demonstrated that the levels of infectious virus declined between days 2 and 3 and further declined the following day. This finding was correlated with a rapid rise of the RNA:TCID_50_ ratio (Fig 4F) and may be explained by the rapid accumulation of defective particles or release of viral RNA from dying cells. Importantly, this finding makes clear that experiments in which only viral RNA levels are measured should be interpreted with caution. Even if we disregard viral RNA levels and only take infectious virus into account, an interesting observation can be made. Feeding on day 2 on a lamb with viremia of 10^5.2^ TCID_50_/ml (lamb 2) resulted in an infection rate of 86%, whereas feeding on day 3 on the other lamb with comparably high viremia (10^5.7^ TCID_50_/ml) resulted in an infection rate of only 18%. Although based on limited data, this observation suggests that some factor in the blood interfered with infection of the mosquitoes on day 3. It is relevant to note that the survival rates of the mosquitoes that had fed on day 3 on both lambs were strikingly lower than those of mosquitoes that had fed on day 2. Specifically, whereas 79% and 78% of the mosquitoes that had fed on day 2 on lamb 1 and 2, respectively, survived until the moment of analysis, only 41% and 50% of the mosquitoes that had fed on day 3 on lambs 1 and 2, respectively, survived until the moment of analysis. The cause of these declines in survival rates is unclear, but may be correlated with the rapidly declining transmission rates.

The infectious threshold for RVFV transmission to mosquitoes was previously proposed to be 10^4.5^ plaque-forming units/ml, corresponding to 10^4.6^ TCID_50_/ml [41]. Based on the levels and duration of viremia in ruminants, transmission of RVFV from these animals to mosquitoes was predicted to occur within a time period of 4 days [41]. Although this theoretical assumption was plausible, our data suggests that a much narrower window of opportunity exists for the virus to infect mosquitoes. However, it is also likely that infectious thresholds differ among mosquito species or even biotypes of *Culex pipiens*, as, for example, transmission rates for West Nile virus differed greatly among biotypes of *Cx*. *pipiens* from The Netherlands [34]. Moreover, the vector competence of *Ae*. *vexans* for RVFV was found to vary greatly depending on the area from which the mosquitoes were collected [11]. Clearly, more research is needed to determine the duration of infectivity of different hosts for specific mosquito species, preferably taking into account environmental and (epi) genetic factors.

Another unexpected observation was that one lamb suddenly succumbed to the infection and that the other lamb had to be euthanized when a humane end point was reached. This high disease burden warranted a thorough post mortem examination of the two lambs. Gross examination revealed severe liver necrosis, which is usually observed in lambs that succumb to RVFV infection, but no other aberrant pathological findings. Examination of the mosquito feeding sites on the inner thighs of the lambs, however, yielded more remarkable results. Apart from extensive haemorrhages in the dermis and an influx of neutrophils and macrophages, IHC staining revealed extensive replication of RVFV in keratinocytes, the endothelium of dermal blood vessels, smooth muscle cells, fat cells and fibroblasts. Macrophages in the dermis were also strongly positive for RVFV antigen, which may have resulted from phagocytosis of virus particles or from replication in these cells. Importantly, similar samples obtained from the other leg of the same animal revealed no signs of RVFV infection, suggesting that this enhanced, localized replication was mediated by the inflammatory response resulting from the mosquito bites. Another striking observation was the margination of RVFV antigen in the blood vessels together with thrombocytes. This may be explained by an interaction of the virus with blood platelets that responded to vascular damage or inflammation. Since the marginated thrombocyte aggregates did not appear to contain fibrin filaments, some component in the mosquito saliva may have interfered with the normal coagulation cascade.

It is relevant to note that the host inflammatory response to mosquito bites was previously shown to enhance the severity of Semliki Forest virus and Bunyamwera virus infection [42]. Furthermore, saliva from *Aedes* mosquitoes was previously shown to modulate RVFV pathogenicity for mice. Interestingly, this was not observed when using salivary gland extracts from *Cx*. *pipiens* [43]. The latter may be a first clue that the influence of mosquito saliva on arbovirus infection varies among virus-host-mosquito combinations.

In conclusion, a future introduction of RVFV into the Netherlands or elsewhere in Europe could result in significant spreading of the virus as Europe, and particularly the Netherlands, is home to high densities of target animals and mosquitoes that have now been shown susceptible to the virus. However, before a proper risk-assessment can be made, additional research is needed on the vector competence of other relevant mosquito species, particularly those of the genus *Aedes*, which can also be abundant certain times of the year and in specific areas [44]. In addition, research on the influence of mosquito bites on the outcome of RVFV infections is warranted not only to gain more fundamental insight into RVF pathogenesis and epidemiology, but also to address the ability of vaccines to protect animals from natural exposure to the virus.
